# Using Health Concept Surveying to Elicit Usable Evidence: Case Studies of a Novel Evaluation Methodology

**DOI:** 10.2196/30474

**Published:** 2022-01-03

**Authors:** Alex Mariakakis, Ravi Karkar, Shwetak N Patel, Julie A Kientz, James Fogarty, Sean A Munson

**Affiliations:** 1 Department of Computer Science University of Toronto Toronto, ON Canada; 2 School of Computer Science & Engineering University of Washington Seattle, WA United States; 3 Department of Human Centered Design & Engineering University of Washington Seattle, WA United States

**Keywords:** mobile health, survey instrument, health screening, health belief model, path analysis, user design, health technology, health intervention technology, digital health, mobile phone

## Abstract

**Background:**

Developers, designers, and researchers use rapid prototyping methods to project the adoption and acceptability of their health intervention technology (HIT) before the technology becomes mature enough to be deployed. Although these methods are useful for gathering feedback that advances the development of HITs, they rarely provide usable evidence that can contribute to our broader understanding of HITs.

**Objective:**

In this research, we aim to develop and demonstrate a variation of vignette testing that supports developers and designers in evaluating early-stage HIT designs while generating usable evidence for the broader research community.

**Methods:**

We proposed a method called *health concept surveying* for untangling the causal relationships that people develop around conceptual HITs. In health concept surveying, investigators gather reactions to design concepts through a scenario-based survey instrument. As the investigator manipulates characteristics related to their HIT, the survey instrument also measures proximal cognitive factors according to a health behavior change model to project how HIT design decisions may affect the adoption and acceptability of an HIT. Responses to the survey instrument were analyzed using path analysis to untangle the causal effects of these factors on the outcome variables.

**Results:**

We demonstrated health concept surveying in 3 case studies of sensor-based health-screening apps. Our first study (N=54) showed that a wait time incentive could influence more people to go see a dermatologist after a positive test for skin cancer. Our second study (N=54), evaluating a similar application design, showed that although visual explanations of algorithmic decisions could increase participant trust in negative test results, the trust would not have been enough to affect people’s decision-making. Our third study (N=263) showed that people might prioritize test specificity or sensitivity depending on the nature of the medical condition.

**Conclusions:**

Beyond the findings from our 3 case studies, our research uses the framing of the Health Belief Model to elicit and understand the intrinsic and extrinsic factors that may affect the adoption and acceptability of an HIT without having to build a working prototype. We have made our survey instrument publicly available so that others can leverage it for their own investigations.

## Introduction

### Overview

There are numerous design decisions beyond the rigor of the information being presented in a health intervention technology (HIT) that can affect how people incorporate the HIT’s guidance into their decision-making [1]. These factors can range from the HIT’s visual appearance [2] and message framing [3,4] to people’s beliefs and psychological traits [5,6]. Late-stage evaluation methods such as A/B field testing and randomized controlled trials are designed to help HIT creators explore the ways in which the aforementioned factors might affect people’s decision-making [7-12]. However, deploying an HIT too early can expose people to numerous risks, such as delays in necessary lifestyle changes, postponed diagnoses, and unwarranted stress. User-centered design also encourages designers to incorporate feedback early and often in their process before reaching these late-stage evaluation methods [13]. Unfortunately, early-stage evaluation and rapid prototyping methods (eg, think-aloud evaluations and paper prototyping) are not as well-suited for eliciting feedback on how people would respond to an HIT’s guidance. Many people assess the credibility of an HIT based on its visual appearance and language [2,14], which may not be fully developed in a low-fidelity prototype. People can also idealize unspecified HIT features to their liking, resulting in a positive but biased evaluation [15]. Even when a prototype is complete, early-stage methods are better suited for identifying *which* features people prefer but not *why* they prefer those features or *how* those features will affect use [16].

In light of these challenges, Klasnja et al [17] called for early-stage evaluation methods that generate *usable evidence*: “empirical findings about the causal effects of [HITs] and how those effects vary with individual differences, context of use, and system design.” Klasnja et al [17] discussed usable evidence in the context of developers and designers who are creating a new HIT; however, there is also a broader need within the research community to generate findings that lead to guidelines and theories. Identifying usable evidence requires an explicit understanding of the causal mechanisms that affect the reception of an HIT [18], which can only be gained by untangling the effects of HIT design decisions and proximal cognitive factors such as beliefs and attitudes.

As a methodological contribution to HIT design research, we propose *health concept surveying*, a variation of vignette testing [19,20] that supports the generation of usable evidence. Health concept surveying is centered on a survey instrument that presents target users with a technology concept in a scenario and then measures the potential impact that HIT design decisions may have on 2 distal outcomes [21,22]: (1) adoption of an HIT, which is a person’s intention of using an HIT, and (2) acceptability of an HIT’s suggestions, which is a person’s willingness to conduct the follow-up actions recommended by the HIT.

The survey instrument also measures proximal cognitive factors as defined by a health behavior change framework (eg, the Health Belief Model, HBM [23,24]). The responses to the survey were analyzed using path analysis to surface causal pathways that inform future research on HITs. As health concept surveying relies on design concepts rather than physical prototypes, HIT creators can be selective about which HIT design characteristics they include to prevent study participants from getting distracted by missing or incomplete features.

We demonstrate the efficacy of health concept surveying using 3 case studies to display its utility for multiple stakeholders. The first 2 case studies show how health concept surveying would be beneficial to a developer or designer invested in a particular HIT, whereas the third case study highlights how researchers could use health concept surveying to test a broader hypothesis across multiple HITs. The case studies are centered on sensor-based health-screening apps—smartphone apps that use on-device sensors such as cameras and microphones to identify the presence of medical symptoms—as this domain is emerging in academia and industry alike [25]. The design decisions that are explored in these case studies include (1) the inclusion of an incentive, (2) the inclusion of visual test result explanations, and (3) the trade-off between the true positive rate and true negative rate.

In summary, our research contributes the following:

1. The health concept surveying method, which uses vignette testing to disentangle the effects of design decisions and proximal cognitive factors on the adoption and acceptability of an HIT.
2. Case studies that show how health concept surveying can be used to benefit specific HIT designs while generating usable evidence for the broader community.
3. A more complex case study that shows how health concept surveying can also support more abstract research to directly contribute to our understanding of HITs.

### Prior Work

Our research is primarily inspired by a collection of commentaries on behavior change technologies (BCTs) by Klasnja et al and Hekler et al [17,26,27]. BCTs aim to persuade a person to change their habits, whereas HITs can include both health-focused BCTs and technologies that provide a 1-time suggestion for a course of action.

In this thread of research, Klasnja et al [26] first recognized that demonstrating behavior change for early-stage BCTs is often “infeasible as well as unnecessary for a meaningful contribution to HCI research” and instead suggest that researchers strive for “a deep understanding of the how and why of the system use by its target users.” They proposed that researchers can work toward such an understanding by tailoring their evaluation methods to the intervention strategies involved in their HIT (eg, self-monitoring, conditioning, and tunneling [28]), which can require the development of new strategies that balance abstraction with contextual relevance [27]. By leveraging behavioral science theories, Klasnja et al [17] suggested that researchers can not only advance their particular intervention but also generate *usable evidence*: “empirical findings about the causal effects of BCTs and how those effects vary with individual differences, context of use, and system design.”

Evaluation methods such as factorial designs [7,8], microrandomized trials [9,10], and single-case experimental designs [11,12] can be used to methodically test hypothesis-driven research; however, these methods are typically considered only after a prototype is sophisticated enough to be put into people’s hands. By using a survey method, health concept surveying allows investigators to include as few or as many details about an HIT as they deem fit. This flexibility of abstraction not only makes the health concept surveying suitable for developers and designers with early-stage HITs but also for researchers as they explore hypotheses around HIT concepts. Health concept surveying also relies on health behavior change frameworks so that researchers can disentangle complicated relationships between factors to generate usable evidence.

### Theory: HBM

Social psychologists have proposed various frameworks to predict, explain, and change health behaviors in matters related to public and personalized health. These frameworks have been applied to topics ranging from smoking cessation and exercise [29] to vaccination [30] and hearing loss prevention [31]. Health behavior change frameworks typically fall into two categories [32]: social cognition models (eg, theory of planned behavior [33] and HBM [23,24]), which use cognitive factors such as beliefs and attitudes as proximal determinants of behavior; and stage models (eg, transtheoretical model [34]), which describe decisions as a sequence of discrete phases.

Survey instruments for applying health concept surveying could be modeled after any of the aforementioned health behavior change frameworks to specify proximal cognitive factors. In this work, we demonstrate health concept surveying with a survey instrument based on the HBM. Researchers have criticized aspects of the HBM, such as its lack of applicability outside of health-related contexts [35,36] and the inconsistency in how different researchers define its constructs [35,37,38]. Nevertheless, we use the HBM because of its specific focus on health interventions, its applicability to both short-term actions and long-term behaviors, and the potential for its constructs to map to actionable feedback for developers, designers, and researchers. By providing a survey instrument that others can use, we hope to provide standardized questions that mitigate inconsistency.

The HBM posits that a person will undergo an action to improve or maintain their health if the perceived barriers to that particular action are outweighed by the perceived seriousness of the health problem, the perceived susceptibility to that health problem, and the perceived benefits of taking action. All of these constructs are affected by modifying variables, that is, demographic information and psychological characteristics that can explain a person’s decision-making. For instance, someone who is well-educated may understand the benefits of early screening, whereas someone who does not have flexible income may view the cost of a screening examination as burdensome. Conceptually, the HBM can be summarized using the following equation:

Modifying variables × (Seriousness + Susceptibility + Benefits – Barriers) + Cues to action = Likelihood of action

Definitions of the HBM constructs according to Urich [39] are provided in Textbox 1.

**The constructs of the Health Belief Model and their definitions.**

**Health Belief Model constructs and definitions**

- Perceived seriousness: a person’s subjective assessment of the severity of the health problem and its potential consequences
- Perceived susceptibility: a person’s subjective assessment of their risk of developing the health problem
- Perceived benefits: a person’s subjective assessment of the value in taking a certain action
- Perceived barriers: a person’s subjective assessment of the obstacles to taking a certain action
- Modifying variables: individual characteristics (demographic and psychosocial) that can affect a person’s perception of a health problem
- Cues to action: internal or external triggers that prompt a certain action

## Methods

### Overview

**Figure 1:**
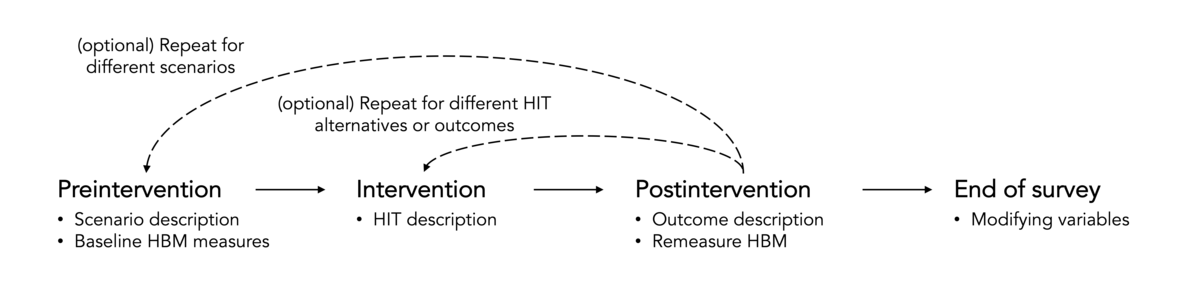
The structure of the survey instrument for health concept surveying comprises four stages: (1) preintervention, (2) intervention, (3) postintervention, and (4) end of survey. HBM: Health Belief Model; HIT: health intervention technology.

Health concept surveying is centered on a survey instrument that allows investigators to measure proximal cognitive factors while manipulating HIT features. In this section, we provide details on the contents of the survey instrument, as illustrated in Figure 1. We illustrated this survey instrument with a concept for a sensor-based health-screening app called SkinCheck, which analyzes the appearance of a person’s mole to determine whether it is cancerous. A complete example of the survey instrument used can be found in Multimedia Appendix 1.

### Survey Design

#### Preintervention

Our survey instrument starts by presenting respondents with a scenario that describes a cue to action related to the health topic of interest for the HIT. Cues to action can include the emergence of symptoms, promotional advertising, or even direct recommendations or prescriptions from a physician. For our example regarding a sinus infection, our prompt was as follows:

You recently noticed a new mole (beauty mark) on your arm that is oddly colored and misshapen. After looking up information online, you worry that you might be developing skin cancer.

After reading the scenario, the respondent is asked to complete an instructional manipulation check (IMC) [40], where they are asked to select the symptoms that are associated with the described medical condition. In addition to checking that the respondent read the scenario, the IMC forces the respondent to spend extra time reflecting on the scenario.

The respondent is then asked a series of questions related to their initial reactions to the scenario according to the constructs of the HBM: *PerceivedSeriousness*, *PerceivedSusceptibility*, *PerceivedBenefits*, and *PerceivedBarriers* (Textbox 2). Each construct has a corresponding question except for *PerceivedSeriousness*, which has 3 questions to account for the various impacts that a health-related issue can have on a person’s life. All responses are recorded on a 7-point scale. The respondent is also asked whether they would take various actions as a series of yes-or-no questions. The respondent is free to take 0, 1, or multiple actions; therefore, we use the variable *ActionType* to keep track of which action corresponds to each response and *ActionTaken* to track whether the respondent would take each action. As people can foresee different *PerceivedBenefits* and *PerceivedBarriers* for various actions, we also ask the respondent to separately rate those questions for each *ActionType*.

**The set of questions that are asked in the pre- and postintervention stages of the health concept surveying survey instrument.**

**Health Belief Model constructs and survey questions**

- Perceived seriousness
  If you had [medical condition] in this scenario, how impactful do you believe it would be on your long-term health?
  If you had [medical condition] in this scenario, how impactful do you believe it would be on your finances?
  If you had [medical condition] in this scenario, how impactful do you believe it would be socially and/or professionally?
- Perceived susceptibility
  How likely do you think you are to have [medical condition] in this scenario?
- Perceived benefits
  How beneficial do you believe each of these actions would be towards helping you recover from your symptoms?
- Perceived barriers
  How easy do you think it would be for you to take each of the following actions to help you recover from your symptoms?
- Action taken
  Given the possibility that you may [have/not have] [medical condition], which of the following actions would you plan to take on the same day as when you discovered your symptoms?

#### Intervention

After the respondents report which actions they would take, they are given information about an HIT that is meant to address the health-related issue described in the scenario. This is where the investigator can choose which details to include about their HIT. Although more details will generally make the HIT concept more concrete and leave less room for uncertainty, the investigator may choose to leave out some information to avoid potential distractions from their primary questions. Our SinusCheck example includes the following text:

A smartphone app named SkinCheck analyzes a picture of a mole to determine whether or not it is cancerous. To use the app, you are asked to take a picture of the mole so that it is clearly visible. The app guides you through taking a picture so that it can see the mole clearly and at a proper distance.

SkinCheck comes with your smartphone by default as part of a new mobile health initiative by [Phone Company]. SkinCheck provides text-based and audio-based instructions to help you perform the test. The app also checks that the test was performed correctly. You can repeat the test until the app determines the image to be “valid.” The results of the test are available instantly.

This example includes a high-level description of the app’s source and functionality; however, it does not include any mockups or screenshots of the app itself. Therefore, an investigator could use this example early in their development process to explore how people would feel about the concept of using an app to detect sinus infections without undue influence from the visuals of the app itself, which could be addressed at a later time.

#### Postintervention Stage

After reading the HIT description, the respondent is asked about their interest in using the HIT on a 7-point scale, which we call *TechnologyInterest*. If the respondent says that they would use the HIT beyond the neutral score, they are taken to pages where they are asked to react to different outcomes in a randomized order. For health-screening apps, our outcomes included positive and negative test results. After each outcome, the respondent is asked to re-evaluate their responses to the questions in Textbox 2. We can determine whether the HIT would have changed the respondent’s plan by comparing *ActionTaken* across the pre- and postintervention stages. This produces a second outcome variable called *ActionChange*, indicating whether the HIT had sufficient influence to change a person’s behavioral intent. Similar to *ActionTaken*, *ActionChange* is recorded for each *ActionType*.

Every HBM construct would ideally be evaluated before and after the intervention to examine how perceptions changed as a result of the intervention. However, doing so can significantly increase the survey length when evaluating multiple versions of an HIT. Therefore, an investigator may choose to remove a postintervention question for a particular HBM if they are confident that their design question is unrelated to it. In such cases, the response from the preintervention stage is propagated through the rest of the respondent’s data, as it is assumed to be constant. We use this modification in our third case study as it has 3 manipulated factors and a mixed factorial study design.

#### End of Survey

At the end of the survey instrument, the respondent is asked for information related to *ModifyingVariables* within the HBM. These questions can capture demographic information (eg, age and access to health care services), psychological properties (eg, risk aversion), or self-assessed expertise in topics related to the HIT (eg, numeracy and familiarity with the medical condition). As the content of the survey itself can provide new information to respondents, some of these questions may be best placed at the beginning of the survey.

#### Design Summary

To summarize, our survey instrument captures two key outcome variables: (1) *TechnologyInterest*, which measures the likelihood that the respondent would use the app on a 7-point scale, and (2) *ActionTaken*, which measures the likelihood that the respondent would take action based on the information available to them at that point in the survey. All respondents would answer questions related to each HBM construct, *TechnologyInterest* and *ActionTaken* in the preintervention stage. Respondents who express sufficient interest in using the HIT are then shown various potential outcomes of the HIT and asked to reanswer the HBM construct and *ActionTaken* questions for each one. The responses to *ActionTaken* in the pre- and postintervention stages are compared for each HIT outcome to form the outcome variable *ActionChange*. *ActionChange* is not recorded for respondents who do not express interest in using the HIT as they never reach the postintervention stage. We use *TechnologyInterest* to project the potential adoption of an HIT, and we use *ActionChange* to project the potential acceptability of an HIT.

### Analysis

We analyzed data from our survey instrument using path analysis, a variant of structural equation modeling that discerns the effects of a set of observable variables on a specified outcome via multiple causal pathways [41]. Path analysis revolves around graphical models called path diagrams, which encode hypothesized causal relationships by using nodes to represent measured constructs and directed edges to represent the relationships between them. Running path analysis produces a model in which each edge is assigned a path coefficient and a corresponding *P* value. We reported standardized path coefficient (*b*), where *b*=0.5 from *X* to *Y*, suggesting that a 1 SD change in *X* produces a 0.5 SD change in *Y*.

The result of path analysis is a model in which each edge in the path diagram is assigned a path coefficient and *P* value. The coefficient is not a correlation coefficient but rather indicates the degree to which one variable influences the other. Chin [42] asserted that meaningful path coefficients have absolute magnitudes >0.2. The models themselves can be assessed according to a variety of fit statistics with no agreed-upon standard [43-45]. We reported two fit statistics: comparative fit index (CFI) and standardized root mean square residual (SRMR). CFI compares the model fit against the fit of an independent model in which the variables are assumed to be uncorrelated, whereas SRMR compares the difference between the residuals of the covariance matrix and the hypothesized covariance model while standardizing for elements with different ranges. Hu and Bentler [46] considered a model fit to be strong when its CFI is ≥0.95 and its SRMR is ≤0.09. The fit statistics are likely to be poor if the path diagram is insufficient for characterizing the relationship between variables (eg, missing nodes or edges) or if the responses to key variables are heavily biased.

It is possible to analyze the data that are gathered with our survey instrument using techniques such as analysis of variance or generalized linear models; however, separate regressions would be needed for each variable with an inbound edge to capture all the causal pathways in the path diagram. Path analysis makes it easier for investigators to contrast the importance of 2 causal relationships as the entire path diagram is processed at once, and the edge weights are directly comparable. Path analysis also makes it possible to characterize the mediated relationships. In other words, the influence of *X* on *Z* via *Y* can be calculated by multiplying the edge weights from *X* to *Y* and from *Y* to *Z*.

**Figure 2:**
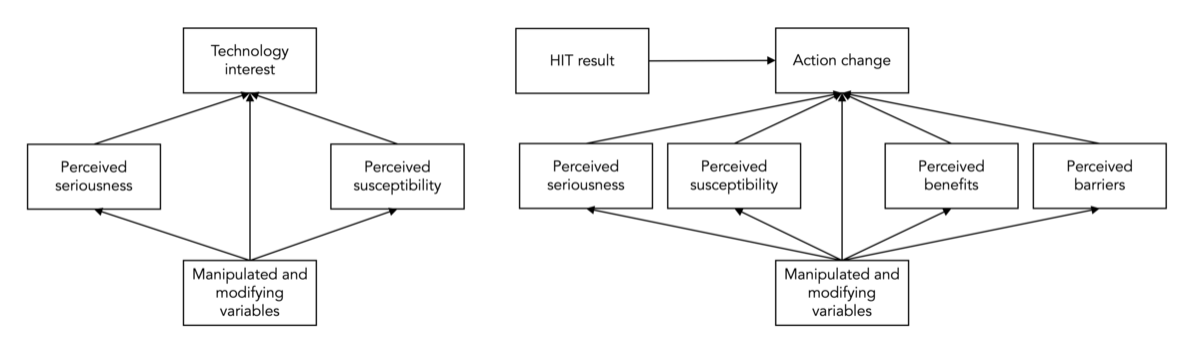
The basic path diagrams used to disentangle the effects that health intervention technology design decisions and user-intrinsic factors have on the measured outcome variables: TechnologyInterest (left) and ActionChange (right). HIT: health intervention technology.

Figure 2 shows the diagram of our outcome variables. *PerceivedSeriousness* is a latent variable that combines the responses to its 3 constituent questions. The more nodes that are in the path diagram, the more complicated the model becomes and the more participants that must be recruited to achieve statistical significance. Therefore, we encourage investigators to remove directed edges between 2 variables if they are confident that the variables are unrelated according to their definition or the investigators’ best judgment. For example, we assume that *TechnologyInterest* is independent of *PerceivedBenefits* and *PerceivedBarriers* as those constructs relate to actions that are unrelated to using the HIT itself. HIT design variables and *ModifyingVariables* should also be added at the investigators’ discretion, with particular focus paid to when they are introduced in the survey instrument. If a design decision affects how the HIT is introduced, the corresponding variable should be added to both path diagrams; however, if the design decision only appears during the intervention stage, the variable should not be included in the *TechnologyInterest* diagram.

We fit the *TechnologyInterest* model to the data from all respondents using their ratings for the HBM constructs in the preintervention stage. Models for *ActionChange* require using data from both the pre- and postintervention stages, therefore limiting the analysis to data from respondents who expressed sufficient interest in using the HIT. Variables such as *ActionType* do not have causal effects but still produce unique entries in the data set. Rather than including these variables in the path diagrams, they are used as grouping factors for multigroup path analysis, a technique in which a model is fit for each group with assumptions about which attributes the models share. As people can have asymmetric reactions to positive and negative test results, we fit separate *ActionChange* models in response to positive (*ActionChangePositive*) or negative (*ActionChangeNegative*) test results when applicable. In each of these cases, we excluded respondents who would have taken the HIT’s *target* action in the preintervention stage. For example, respondents who would have taken action in the preintervention stage were excluded from the model because a positive test result would not be needed to convince them to take action.

## Results

### Overview

To demonstrate the flexibility of our method in a series of case studies, we first had to create a variety of prompts for plausible health-related scenarios and sensor-based health-screening apps. We selected three scenarios based on their plausibility and the different reactions we expected them to elicit: (1) a scenario involving pink eye, which represents a *common* medical condition; (2) a scenario involving skin cancer, which represents a *serious* medical condition; and (3) a scenario involving halitosis, which represents a *stigmatizing* medical condition. Multimedia Appendix 2 [47-58] explains the formative study by which these categories and scenarios were selected.

We used these scenarios to generate 3 case studies that highlighted the diverse ways in which health concept surveying can be used. Our first 2 case studies, which are centered around the skin cancer scenario described in the previous section, illustrate how an HIT developer or designer can use health concept surveying to decide whether to include a feature in their HIT. Our third case study relies on all 3 scenarios to demonstrate how a human–computer interaction (HCI) researcher can use health concept surveying to elicit usable evidence without focusing on a single HIT. We restricted our investigation to a single *ActionType* (scheduling an appointment) for brevity; however, we featured multigroup path analysis in case study 3 to account for its mixed factorial design and demonstrate the expressivity of our method.

### Recruitment

As our case studies were centered on health-screening apps, we recruited participants from the general population without any inclusion or exclusion criteria regarding their experiences with the relevant medical conditions. We sent calls for participation through Facebook, Reddit, and a mailing list within the University of Washington’s Institute of Translational Health Sciences, a center sponsored by the National Institutes of Health’s Clinical and Translational Science for connecting clinicians, patients, and other communities throughout the northwest United States. We excluded respondents who were aged <18 years or did not own a smartphone. Respondents electronically consented before viewing any of the survey materials. Respondents who completed the survey were eligible for a raffle in which 1 in 20 people would win a US $20 Amazon gift card. We used this recruitment strategy for all 3 of our case studies with approval from the University of Washington’s Institutional Review Board (#00003540). Participants were restricted from taking part in multiple case studies to avoid any potential carryover effects or biases (eg, learning and fatigue).

### Case Study 1: Incentivizing Clinical Visits

#### Overview

Our first case study investigated whether the inclusion of a wait time guarantee provides a sufficient incentive for people who would not normally seek medical attention to change their minds and get treatment. We explored this question in the context of our *serious* medical condition scenario regarding skin cancer. We recruited 54 respondents for this case study, and their demographic information can be found in Table 1.

**Table 1:** Demographic information for the people who completed the survey in case study 1 (N=54).

Survey demographics		Values, n (%)
**Source**		
	Facebook	6 (11)
	ITHS^a^	48 (89)
**Gender**		
	Female	41 (76)
	Male	11 (20)
	Gender variant/nonconforming	2 (4)
**Age (years)**		
	18-24	31 (57)
	25-34	13 (24)
	35-44	7 (13)
	45-54	1 (2)
	55-64	2 (4)
**Smartphone operating system**		
	iOS	34 (63)
	Android	20 (37)
**Self-reported smartphone experience**		
	Expert or advanced	32 (59)
	Intermediate	21 (39)
	Novice or beginner	1 (2)

^a^ITHS: Institute of Translational Health Sciences.

#### Study Design

**Figure 3:**
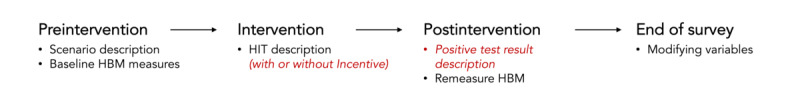
The survey structure for case study 1. The inclusion of an incentive in the health intervention technology description was randomized across respondents. HBM: Health Belief Model; HIT: health intervention technology.

Figure 3 shows the survey design used in this study. We modified the intervention stage so that respondents were shown 1 of the 2 app descriptions at random. Half of the respondents read the SkinCheck description presented in the *Methods* section, whereas the other half saw the same description with the addition of the following text to describe a wait time incentive:

Because of their mobile health initiative, [Phone Company] has an exclusive partnership with dermatologists across the country. People who have a questionable mole on their skin according to SkinCheck are given a promotional code that they can redeem at their local dermatologist to guarantee a wait time no longer than 10 minutes.

As the incentive was intended to make it easier for a person to see a clinician, we only asked respondents about how they would react to a positive test result. The study had a single-factor between-subjects design with the inclusion of an *Incentive* as the factor of interest. As our lone modifying variable, we asked respondents to rate how quickly they thought they would be able to see their physician as we hypothesized that people who did not have convenient access to a clinician would be more influenced by the incentive. We called this variable *Convenience*, and it was measured on a 7-point scale. *Incentive* and *Convenience* were connected to all major HBM constructs and outcome variables in our path diagrams.

This survey had a completion rate of 83% when we accounted for respondents who ended the survey early, satisfied the exclusion criteria, or did not correctly answer the IMC embedded in the survey. Ignoring 2 cases where respondents took more than an hour-long break while completing the survey, the median survey completion time was 8 minutes.

#### TechnologyInterest

Most respondents expressed interest in using the SkinCheck app. Of the respondents who completed the survey, 54% (29/54) gave the highest rating possible for *TechnologyInterest*, 19% (10/54) gave the second-highest rating, 13% (7/54) gave the third-highest rating, and the remaining 15% (8/54) gave ratings that were either neutral or worse. The heavy bias in *TechnologyInterest* meant that a strong model fit could not be found for this outcome variable (CFI=0.839; SRMR=0.131).

#### ActionChangePositive

Table 2 shows the causal path coefficients for the *ActionChangePositive* model fit. Across all respondents who expressed sufficient interest in using the app, 52% (24/46) said they would not have scheduled an appointment before using the app. After being presented with a positive test result, 75% (18/24) changed their mind: 56% (10/18) were shown an incentive and 44% (8/18) were not.

**Table 2:** Path analysis coefficients for ActionChangePositive in case study 1 (CFI^a^=0.951; SRMR^b^=0.079).^c^

Variables	ActionChange	Seriousness	Susceptibility	Benefits	Barriers
AppResult	6.874^d^	−0.002	0.636^e^	0.024	−0.035
Incentive	1.138	−0.406	0.275	0.598	−0.361^e^
Convenience	0.128^e^	−0.492	0.055	−0.168	−0.384^f^
Seriousness	−0.005	N/A^g^	N/A	N/A	N/A
Susceptibility	0.482^e^	N/A	N/A	N/A	N/A
Benefits	0.402^e^	N/A	N/A	N/A	N/A
Barriers	−0.791^d^	N/A	N/A	N/A	N/A

^a^CFI: comparative fit index.

^b^SRMR: standardized root mean square residual.

^c^The columns indicate dependent variables, whereas the rows indicate independent variables.

^d^*P*<.001.

^e^*P*<.05.

^f^*P*<.01.

^g^N/A: not applicable.

The model fit had a large positive coefficient from *AppResult* to *ActionChangePositive* (*b*=6.874; *P*<.001), which was expected because respondents had to see a test result to change their opinion. There was also a strong positive coefficient from *AppResult* to *PerceivedSusceptibility* (*b*=0.636; *P*<.05), which supported our intuition that a positive test result should increase a person’s perceived likelihood of having skin cancer. *ActionChangePositive* was heavily influenced by most of the HBM constructs. The strongest influence came from *PerceivedBarriers* (*b*=−0.791; *P*<.001), which was negative as barriers make it more difficult for a person to be able to take action.

Although there were strong coefficients from *Incentive* to all HBM constructs, the only statistically significant relationship was from *Incentive* to *PerceivedBarriers* (*b*=−0.361; *P*<.05). The fact that there is a negative coefficient between the 2 supported our expectation that the incentive would diminish the obstacles that respondents would foresee in the scenarios. Combining this finding with the strong negative coefficient from *PerceivedBarriers* to *ActionChangePositive* implies that *Incentive* had a strong positive effect on *ActionChangePositive* mediated by *PerceivedBarriers*. However, the coefficient from *Convenience* to *PerceivedBarriers* (*b*=−0.384; *P*<.01) is slightly larger in magnitude than that from *Incentive*, which indicates that the incentive was somewhat less important than the convenience of getting to a clinician in the first place. Further investigation into our data set revealed that most individuals who decided to take action after seeing a positive test result paired with an incentive gave less than a neutral rating for *Convenience*; the *Convenience* ratings for the individuals who were not shown an incentive were more evenly distributed.

### Case Study 2: Presentation of Results

#### Overview

Our second case study investigated how the presentation of test results may influence a person’s decision-making. We examined whether the inclusion of visuals that explain an algorithm’s decision would engender more trust in an app’s test result. As before, we explored this question in the context of our *serious* medical condition scenario regarding skin cancer. We recruited 54 respondents for this case study, and their demographic information can be found in Table 3.

**Table 3:** Demographic information for the people who completed the survey in case study 2 (N=54).

Survey demographics		Values, n (%)
**Source**		
	Facebook	3 (6)
	ITHS^a^	51 (94)
**Gender**		
	Female	45 (83)
	Male	8 (15)
	Undisclosed	1 (2)
**Age (years)**		
	18-24	34 (63)
	25-34	13 (24)
	35-44	2 (4)
	45-54	2 (4)
	55-64	2 (4)
	Undisclosed	1 (2)
**Smartphone operating system**		
	iOS	39 (72)
	Android	15 (28)
**Self-reported smartphone experience**		
	Expert or advanced	28 (52)
	Intermediate	26 (48)

^a^ITHS: Institute of Translational Health Sciences.

#### Study Design

**Figure 4:**
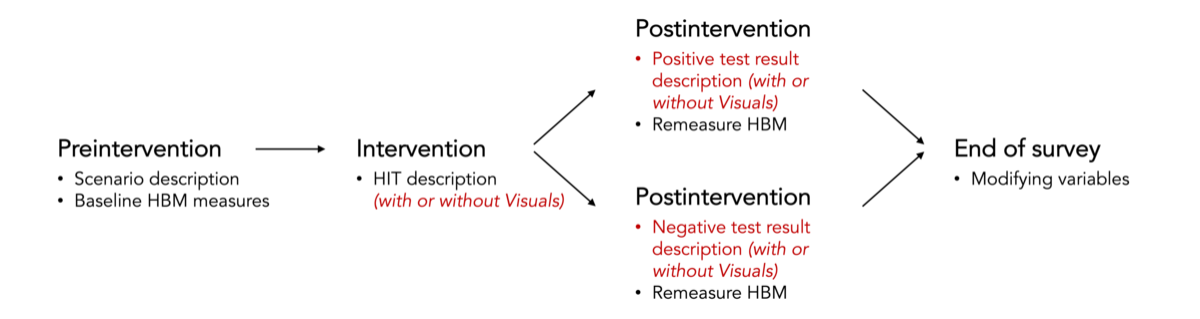
The 2 possible interface options that respondents could have been shown in case study 2 when presented with a positive test result: the interface with text descriptions only (left) and the interface with text and visuals to illustrate how the results were obtained (right). The interfaces were primarily inspired by the DermoScreen app by Wadhawan et al [47]. HBM: Health Belief Model; HIT: health intervention technology.

**Figure 5:**
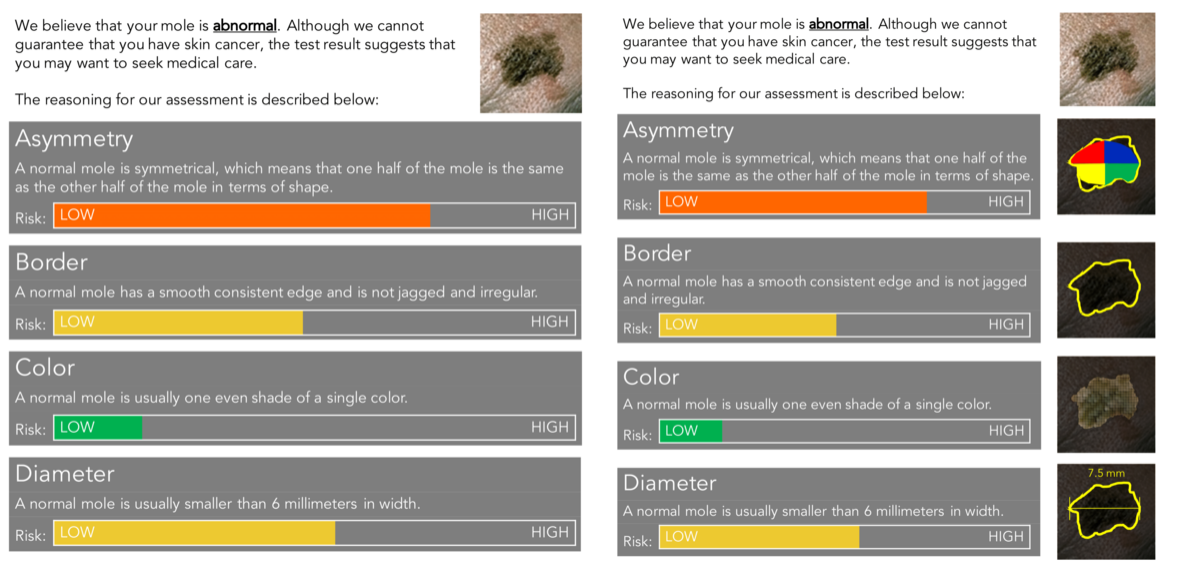
The two possible interface options that respondents could have been shown in Case Study 2 when presented with a positive test result: (left) the interface with text descriptions only and (right) the interface with text and visuals to illustrate how the results were obtained. The interfaces were primarily inspired by Wadhawan et al.’s [47] DermoScreen app.

Figure 4 shows the survey design used in this study. We modified the postintervention stage so that respondents would be asked to react to both positive and negative test results. Instead of explaining the test result in a paragraph, as in the previous case study, respondents were shown 1 of 2 result screen concepts, illustrated in Figure 5 [47], at random. Both screens were derived from the DermoScreen app by Wadhawan et al [47], which explains diagnostic decisions using the ABCD rule of dermatoscopy [59].

The study had a single-factor between-subjects design with the inclusion of *Visuals* as the factor of interest. As our lone modifying variable, we asked respondents about their highest level of education as we hypothesized that reading comprehension would affect their understanding of the visualizations; we called this variable *Education*. *Visuals* and *Education* were connected to all major HBM constructs and outcome variables in our path diagrams.

This survey had a completion rate of 82% when we accounted for respondents who ended the survey early, satisfied the exclusion criteria, or did not correctly answer the IMC embedded in the survey. Ignoring 1 case when a respondent took more than an hour-long break while completing the survey, the median survey completion time was 9 minutes.

#### TechnologyInterest

Most respondents expressed interest in using the SkinCheck app. Of the respondents who completed the survey, 56% (30/54) gave the highest rating possible for *TechnologyInterest*, 19% (10/54) gave the second-highest rating, 15% (8/54) gave the third-highest rating, and the remaining 11% (6/54) gave ratings that were either neutral or worse. The heavy bias in *TechnologyInterest* meant that a strong model fit could not be found for this outcome variable (CFI=0.874; SRMR=0.096).

#### ActionChangePositive

Across all respondents who expressed sufficient interest in using the app, there were 56% (27/48) of cases when people said that they would not have acted before using the app. After being presented with a positive test result, 78% (21/27) changed their mind: 38% (8/21) were shown visuals and 62% (13/21) were not. The inclusion of explanations clearly had an impact on people’s reaction to the positive test result as the frequency of *ActionChangePositive* was much higher than in the first case study. In fact, there were so few cases when people did not act even after seeing a positive test result that there was not enough data to generate a meaningful model fit (CFI=0.644; SRMR=0.197).

#### ActionChangeNegative

Table 4 shows the causal path coefficients for the *ActionChangeNegative* model fit. Across all respondents who expressed sufficient interest in using the app, there were 52% (25/48) of cases when people said that they would have acted before using the app. After being presented with a negative test result, 48% (12/25) changed their mind: 50% (6/12) were shown visuals and 50% (6/12) were not.

As *ActionChangeNegative* is positive when a person is swayed to not act in the postintervention stage, we expected many of the path coefficients to be negated relative to those observed with *ActionChangePositive* in the first case study. This expectation was confirmed in a couple of instances. First, the negative coefficient from *AppResult* to *PerceivedSusceptibility* (*b*=−0.222; *P*<.05) confirmed our intuition that a negative test result should decrease a person’s belief that they had skin cancer in this scenario. Second, the negative coefficient from *PerceivedSeriousness* to *ActionChangeNegative* (*b*=−0.220; *P*<.05) showed that people who were not as concerned about skin cancer were more likely to change their course of action.

As we hypothesized, including additional information in the form of visuals strengthened respondents’ confidence in their test results. This was reflected in the negative coefficient from *Visuals* to *PerceivedSusceptibility* (*b*=−0.961; *P*<.01); when shown a negative test result with visuals, respondents were less likely to believe they had skin cancer. However, *PerceivedSusceptibility* was not influential on *ActionChangeNegative* (*b*=−0.056, not significant); therefore, the inclusion of visuals had neither a direct nor indirect effect on a person’s decision to change their action. We also found that *Education* was not an influential factor for any of the measured constructs or outcome variables.

**Table 4:** Path analysis coefficients for ActionChangeNegative in case study 2 (CFI^a^=0.961; SRMR^b^=0.078).^c^

Variables	ActionChange	Seriousness	Susceptibility	Benefits	Barriers
AppResult	6.588^d^	0.000	−0.222^e^	0.000	−0.342
Visuals	0.231	0.235	−0.961^f^	−0.591	−0.610
Education	0.087	0.086	0.037	0.107	0.055
Seriousness	−0.220^e^	N/A^g^	N/A	N/A	N/A
Susceptibility	−0.056	N/A	N/A	N/A	N/A
Benefits	−0.233^e^	N/A	N/A	N/A	N/A
Barriers	0.223	N/A	N/A	N/A	N/A

^a^CFI: comparative fit index.

^b^SRMR: standardized root mean square residual.

^c^The columns indicate dependent variables, whereas the rows indicate independent variables.

^d^*P*<.001.

^e^*P*<.05.

^f^*P*<.01.

^g^N/A: not applicable.

### Case Study 3: Accuracy

#### Overview

In our third and final case study, we explored the trade-off between false positives and false negatives across medical conditions of varying concern and severity. We leveraged all three of our scenarios (*common*, *serious*, and *stigmatizing*) in a mixed factorial study design, thus necessitating more participants. In total, 263 respondents completed the survey from start to finish, and their demographic information can be found in Table 5.

**Table 5:** Demographic information for the people who completed the survey in case study 3 (N=263).

Survey demographics		Values, n (%)
**Source**		
	Facebook	16 (6.1)
	ITHS^a^	240 (91.3)
	Reddit	3 (1.1)
	Other	4 (1.5)
**Gender**		
	Female	202 (76.8)
	Male	45 (17.1)
	Transgender male	5 (1.9)
	Gender variant/nonconforming	7 (2.7)
	Self-identify	1 (0.4)
	Undisclosed	3 (1.1)
**Age (years)**		
	18-24	145 (55.1)
	25-34	84 (32)
	35-44	17 (6.5)
	45-54	8 (3.1)
	55-64	3 (1.1)
	≥65	3 (1.1)
	Undisclosed	3 (1.1)
**Smartphone operating system**		
	iOS	170 (64.6)
	Android	93 (35.4)
**Self-reported smartphone experience**		
	Expert or advanced	146 (55.5)
	Intermediate	115 (43.7)
	Novice or beginner	2 (0.8)

^a^ITHS: Institute of Translational Health Sciences.

#### Study Design

**Figure 6:**
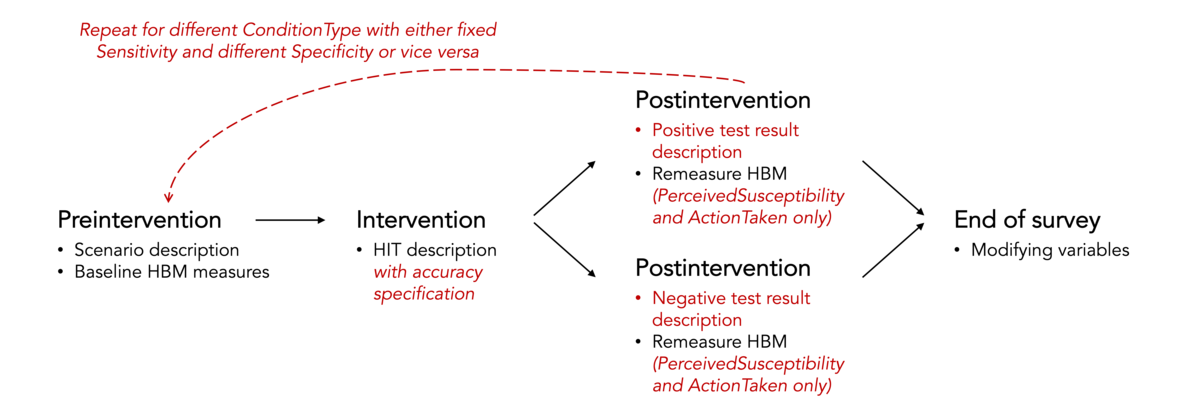
The survey structure for case study 3. Respondents were shown 3 different health intervention technologies (HITs)—1 for each ConditionType. The 3 HITs either had the same sensitivity and varied in specificity or had the same specificity and varied in sensitivity. Respondents were asked to react to positive and negative app results in a randomized order. Only PerceivedSusceptibility and ActionChange were remeasured in the postintervention stages to shorten the survey length. HBM: Health Belief Model; HIT: health intervention technology.

Figure 6 shows the survey design for this study, which required changes in both the intervention and postintervention stages. The app descriptions included information about their classification sensitivity and specificity; sensitivity refers to the proportion of people who are correctly identified as having the medical condition out of all those who have it, whereas specificity refers to the proportion of people who are correctly identified as not having the medical condition out of all those who do not have it. Because the general public is more adept at reasoning about counts than fractional quantities [60], the sensitivity and specificity rates were presented with counts and icon arrays. An example of the accompanying text is provided as follows:

Out of every 100 people who have a sinus infection, SinusCheck correctly told 65 people that they had a sinus infection.

Out of every 100 people who do not have a sinus infection, SinusCheck correctly told 80 people that they did not have a sinus infection.

The survey was used in a 3×3×3 mixed factorial study design. Each respondent read all three scenarios—pink eye (*common*), skin cancer (*serious*), and halitosis (*stigmatizing*)—making *ConditionType* a within-subjects factor. The presentation order of the scenarios was counterbalanced across all subjects. A total of 3 equally spaced levels of sensitivity and specificity were investigated—65%, 80%, and 95%—producing 9 possible combinations that described the overall accuracy of the apps. Each app for each respondent was assigned 1 of the 9 combinations at random, making *Sensitivity* and *Specificity* between-subjects factors. Although there is an inherent trade-off between sensitivity and specificity when the underlying classification algorithm is fixed, we treated them as independent variables in our study design and analyses. As respondents had to go through multiple scenarios, we shortened the survey by only remeasuring *PerceivedSusceptibility* and *ActionTaken* during the postintervention stage. The other major HBM constructs were not remeasured as we assumed that they should not be influenced by app accuracy. As such, *Sensitivity* and *Specificity* were connected to *PerceivedSusceptibility* and the outcome variables in our path diagrams, and *ConditionType* was used as the grouping variable for multigroup path analysis.

This survey had a completion rate of 73% when we accounted for respondents who ended the survey early, satisfied the exclusion criteria, or did not correctly answer the IMCs embedded in the survey. Ignoring 14 cases when respondents took more than an hour-long break while completing the survey, the median survey completion time was 16 minutes.

#### TechnologyInterest

Table 6 shows the causal path coefficients for the *TechnologyInterest* model fit. The path coefficients from *Sensitivity* and *Specificity* to *TechnologyInterest* were sizable and positive across all scenarios, confirming that higher accuracy made the apps more attractive. In fact, the effect was so strong that those coefficients were larger and more statistically significant than those from the HBM constructs. This suggests that respondents were willing to use these apps regardless of their perception of the medical conditions’ threat as long as they knew that the app was accurate.

**Table 6:** Path analysis coefficients for ActionChangeNegative in case study 3 (CFI^a^=0.956; SRMR^b^=0.075).^c^

Variables	Technology Interest		
	Common	Serious	Stigmatizing
Seriousness	−0.120	0.101	0.129
Susceptibility	0.206^d^	0.120^e^	0.104
Sensitivity	0.416^f^	0.357^f^	0.268^d^
Specificity	0.461^f^	0.300^d^	0.292^f^

^a^CFI: comparative fit index.

^b^SRMR: standardized root mean square residual.

^c^The columns indicate dependent variables, whereas the rows indicate independent variables.

^d^*P*<.01.

^e^*P*<.05.

^f^*P*<.001.

Overall accuracy was most valued for the *common* condition (*Sensitivity*: *b*=0.416, *P*<.001; *Specificity*: *b*=0.461, *P*<.001), followed by the *serious* (*Sensitivity*: *b*=0.357, *P*<.001; *Specificity*: *b*=0.300, *P*<.01) and *stigmatizing* (*Sensitivity*: *b*=0.268, *P*<.01; *Specificity*: *b*=0.292, *P*<.001) conditions. Respondents preferred apps with higher accuracy; however, they attributed more importance to sensitivity or specificity depending on the scenario; they placed more importance on specificity for the *common* and *stigmatizing* conditions, whereas they placed more importance on sensitivity for the *serious* condition. Sensitivity and specificity were treated independently in our analysis; therefore, these results do not account for the fact that improving one metric often requires compromising the other during the development of the classification model. Nevertheless, this result suggests that respondents had an inherent knowledge about the notion of prevalence and how it relates to diagnostic decision-making. *Common* and *stigmatizing* conditions are typically prevalent; therefore, prioritizing specificity may indicate that respondents were eager to use an app’s test result to *rule out* having the condition. *Serious* conditions are often less prevalent; therefore, prioritizing sensitivity may indicate that respondents were eager to *rule in* having the condition.

#### ActionChangePositive

Table 7 shows the causal path coefficients for the *ActionChangePositive* model fit. Across all respondents who expressed sufficient interest in using any of the 3 apps, there were 56.5% (359/635) of cases when respondents said that they would not have taken action before using the app. After being presented with a positive test result, 46.2% (166/359) changed their mind: 28.3% (47/166) in the *common* scenario, 42.2% (70/166) in the *serious* scenario, and 29.5% (49/166) in the *stigmatizing* scenario.

**Table 7:** Path analysis coefficients for ActionChangePositive in case study 3 (CFI^a^=0.981; SRMR^b^=0.078).^c^

Variables	Common			Serious			Stigmatizing	
	ActionChange	Susceptibility	ActionChange		Susceptibility	ActionChange		Susceptibility
AppResult	6.962^d^	0.398^d^	6.521^d^		1.518^d^	5.900^d^		0.474^e^
Sensitivity	−0.262	0.283^e^	0.011		0.014	−0.004		0.204^f^
Specificity	−0.095	0.095	−0.212		−0.093	0.033		−0.032
Seriousness	−0.149	N/A^g^	−0.124		N/A	0.168		N/A
Susceptibility	0.426^e^	N/A	0.509^d^		N/A	0.474^d^		N/A
Benefits	0.226^e^	N/A	0.273		N/A	0.055		N/A
Barriers	−0.137	N/A	−0.061		N/A	−0.038		N/A

^a^CFI: comparative fit index.

^b^SRMR: standardized root mean square residual.

^c^The columns indicate dependent variables, whereas the rows indicate independent variables.

^d^*P*<.001.

^e^*P*<.01.

^f^*P*<.05.

^g^N/A: not applicable.

Although there were large positive coefficients from *AppResult* to *ActionChangePositive* and *PerceivedSusceptibility* across all scenarios, the magnitude and significance of those coefficients varied across the medical conditions. The coefficient from *AppResult* to *PerceivedSusceptibility* for the *Serious* condition (*b*=1.518; *P*<.001) was 3 times as large and more significant than the corresponding coefficients for the *common* (*b*=0.398; *P*<.001) and *stigmatizing* (*b*=0.474; *P*<.01) conditions. Again, this result suggests that respondents may have been eager to use the positive test result from an app to *rule in* having a *serious* condition.

*Sensitivity* had a significant positive effect on *PerceivedSusceptibility* for the *common* (*b*=0.283; *P*<.001) and *stigmatizing* (*b*=0.204; *P*<.05) conditions. There were also significant positive coefficients between *PerceivedSusceptibility* and *ActionChangePositive* in those scenarios (common: *b*=0.426, *P*<.01; stigmatizing: *b*=0.474, *P*<.001), which means that *Sensitivity* had a strong effect on *ActionChangePositive* mediated by *PerceivedSusceptibility* in those scenarios. In other words, respondents were more likely to be convinced to change their course of action after seeing a positive test result when the app had a higher sensitivity. *Sensitivity* did not have a significant effect on *PerceivedSusceptibility* for the *serious* condition (*b*=0.014, not significant), implying that respondents were equally willing to accept a positive test result across the presented sensitivity rates in that scenario. *Specificity* did not have a statistically significant effect on either *ActionChangePositive* or *PerceivedSusceptibility* for any of the scenarios. Although sensitivity corresponds to a test’s true negative rate, this finding is still notable as sensitivity affects how a positive test result should be interpreted according to Bayesian statistics.

#### ActionChangeNegative

Table 8 shows the causal path coefficients for the *ActionChangeNegative* model fit. We note that this model had borderline significance according to our fit statistics, satisfying the threshold for SRMR but not for CFI. Across all respondents who expressed sufficient interest in using any of the 3 apps, there were 41.9% (266/635) of cases when respondents said that they would have taken action before using the app. After being presented with a negative test result, 51.9% (138/266) changed their minds: 39.1% (54/138) in the *common* scenario, 37.7% (52/138) in the *serious* scenario, and 23.2% (32/138) in the *stigmatizing* scenario.

**Table 8:** Path analysis coefficients for ActionChangeNegative in case study 3 (CFI^a^=0.925; SRMR^b^=0.077).^c^

Variables	Common			Serious			Stigmatizing	
	ActionChange	Susceptibility	ActionChange		Susceptibility	ActionChange		Susceptibility
AppResult	6.230^d^	−1.999^d^	6.144^d^		−0.970^d^	6.833^d^		−2.191^d^
Sensitivity	0.022	−0.196	−0.022		−0.026	0.003		0.056
Specificity	−0.103^e^	0.094	0.063		−0.185^e^	−0.192		−0.119
Seriousness	−0.451^e^	N/A^f^	−0.182		N/A	−0.169		N/A
Susceptibility	−0.311^d^	N/A	−0.358^d^		N/A	−0.212^d^		N/A
Benefits	0.001	N/A	0.125		N/A	−0.196		N/A
Barriers	0.105	N/A	0.083		N/A	−0.176		N/A

^a^CFI: comparative fit index.

^b^SRMR: standardized root mean square residual.

^c^The columns indicate dependent variables, whereas the rows indicate independent variables.

^d^*P*<.001.

^e^*P*<.05.

^f^N/A: not applicable.

As with the model fit for *ActionChangePositive*, there were statistically significant coefficients from *AppResult* to *PerceivedSusceptibility* and *ActionChangeNegative*; however, their magnitude varied across *ConditionType*. The coefficients from *AppResult* to *PerceivedSusceptibility* for the *common* (*b*=−1.999; *P*<.001) and *stigmatizing* (*b*=−2.191; *P*<.001) conditions were nearly double the corresponding coefficient for the *serious* condition (*b*=−0.970; *P*<.001), suggesting that respondents may have been eager to use the negative test result from those apps to *rule out* having those conditions.

In the *serious* condition scenario, significant negative coefficients were found from *specificity* to *PerceivedSusceptibility* (*b*=−0.185; *P*<.001) and *PerceivedSusceptibility* to *ActionChangeNegative* (*b*=−0.358; *P*<.001). This combination of results implies that respondents were more likely to be convinced to change their course of action after seeing a negative test result when the app had a higher specificity. *Specificity* did not have a significant effect on *PerceivedSusceptibility* in either the *common* (*b*=0.094, not significant) or the *stigmatizing* (*b*=−0.119, not significant) conditions, indicating that respondents were equally willing to accept a negative test result across the presented specificity rates in those scenarios. *Sensitivity* did not have a statistically significant effect on either *ActionChangeNegative* or *PerceivedSusceptibility* for any of the scenarios, which mirrors the earlier findings with respect to *specificity* and positive test results.

## Discussion

### Principal Findings

We sought to develop a low-burden method for projecting the adoption and acceptability of an HIT, given different design variations. Our contribution toward this goal—the health concept surveying method—supports HIT investigators in advancing their own HITs while generating usable evidence for the broader research community. Our 3 case studies highlight the different types of actionable feedback and usable evidence that can be elicited using our survey instrument without deploying a working HIT prototype.

Our first case study showed that a wait time incentive might support some individuals in overcoming barriers that could prevent them from visiting a dermatologist. However, many participants said that they would be persuaded to act without an incentive. This result suggests that HIT developers in this scenario may want to consider additional messaging that targets other facets of the HBM, such as the perceived susceptibility people have to skin cancer or the perceived benefits of seeking a second opinion. We also found that access to convenient health care was an important factor in people’s decision-making; therefore, developers in this scenario may want to examine whether this is an important issue to address for their target audience.

Our second case study showed that SkinCheck’s baseline explanation could be convincing enough to sway a person to visit a clinician when they received a positive test result. The inclusion of visuals increased individuals’ trust in negative test results; however, this was not enough to significantly affect people’s decision-making. In fact, we found that the main driving factor for people who decided not to act after seeing a negative test result was the perceived seriousness of skin cancer. This presents an interesting challenge for HIT designers. Lowering a person’s concern about the severity of a medical condition could have major consequences, including the fact that they may ignore a positive test result later on because of their newfound understanding of the condition. Instead, HIT designers in this scenario may want to consider using a language that diminishes a person’s short-term concerns but encourages repeated testing in the near future.

Our third case study suggests that researchers may want to consider the trade-off between sensitivity and specificity in the context of their target medical condition. Kay et al [61] elicited similar findings through a survey instrument they created to understand the acceptability of precision and recall across various sensor-based technologies. In an example involving a home alarm system, they showed that participants were more willing to accept false alarms when the system had a benign intervention (eg, contacting the homeowners via SMS text message) than when the system had an intrusive intervention (eg, automatically alerting the police). To improve the user experience that people have with a classifier-based application, HIT developers may consider adjusting the final decision threshold of their classifier to minimize errors that people are more prone to believe. However, doing so may serve as an expedient solution to the greater challenge of helping ordinary people with Bayesian reasoning.

### Other Design Decisions for Exploration

We explored the influence of 3 different design choices on outcomes relevant to HITs (incentives, results presentation, and accuracy trade-offs); however, there are many others that would be interesting to explore in future work. One of those factors would be the HIT’s price. When we first piloted our studies, we stated that the apps could be purchased on app stores for US $0.99. We selected such a low cost as we were worried that a free app would appear illegitimate; however, an expensive app would diminish interest to the point that we would not receive feedback from respondents. However, some of the respondents in our pilot study felt that a US $0.99 app appeared *less legitimate than a free app* and *cheap*, so we instead crafted scenarios in which the app was already included on the respondents’ phones. The economics research community has debated the relationship between price and perceived product quality; some researchers argue that there is generally a positive correlation between price and quality [62], whereas others argue that the 2 are only correlated under contrived scenarios [63].

Another factor that influences the perceived quality of technology is endorsements [64]. App stores, smartphone manufacturers, special interest groups, and physicians can all endorse technologies, serving as a *seal of approval* that may imbue an HIT with legitimacy. A limitation of our survey instrument is that it is difficult to convey an endorsement to respondents without explicitly drawing the respondents’ attention to it. Endorsements can appear in many places—commercials, supplemental materials, or websites—that may not be as conspicuous as mentioning would be done in the survey. Determining a more natural way of introducing endorsements within health concept surveying could be a potential avenue for future work.

### Alternative and Complementary Approaches

Health concept surveying is one of many early-stage quantitative research methods that developers and designers can use to further their understanding of HITs. Conjoint analysis and discrete choice experiments elicit preferences by asking participants to pick between options with 1 or many feature variations in a head-to-head comparison [65]. Another relevant technique is judgment analysis [66,67], where feature preferences are gathered by comparing the decisions that participants make in hypothetical scenarios against a predefined oracle or reference group. All of these methods have been used to investigate people’s decision-making in the health domain [68-70]; however, health concept surveying has the advantage of being designed so that investigators can project both the adoption of an HIT and the acceptability of an HIT’s suggestions. By accounting for intrinsic and extrinsic factors that can influence these distal outcomes, health concept surveying is able to elicit usable evidence that HIT developers and designers can apply to their own HITs.

We view health concept surveying as being complementary to qualitative research methods such as focus group interviews, which give participants the chance to verbalize their thoughts and decision-making in a richer way than what can be gathered through a survey. That said, health concept surveying is far more efficient to scale. Focus groups must be run with 5 to 10 participants at a time, and investigators must often conduct multiple sessions to reach diverse populations or gather feedback on new design iterations. Each new session incurs an additional time investment for both the interviews and the qualitative analyses, making focus groups difficult to scale as an HIT evolves. In addition, focus groups have known confounds such as group-think or dominance by 1 or 2 individuals, even in light of techniques to mitigate these confounds [71]. With health concept surveying, adding more participants simply requires distributing the survey to more people and then rerunning the same analysis code as before, imposing no additional burden beyond what is required for recruitment. Health concept surveying also helps investigators systematically analyze the influence of all the variables involved in people’s decision-making, which can otherwise be difficult for participants to articulate and for investigators to translate into usable evidence. We hope that our work inspires HCI researchers to explore how people can incorporate psychological frameworks into other evaluation techniques.

### Limitations

Several psychological frameworks for explaining behavior rely on the belief that intention is a strong predictor of behavior. The correlation between intention and behavior has been supported by research on health-related topics such as dieting [72], physical activity [73,74], and weight loss [75]. Nevertheless, people’s behavioral intentions or expected actions do not always lead to completing the action because of the emergence of unforeseen barriers or changing beliefs over time. Psychologists have called this phenomenon *the intention–behavior gap* [76,77]. This potential disconnect exists in most early-stage evaluation methods; however, the gap may be particularly relevant to health concept surveying as intention in scenario-based study designs may not translate to real-world actions, and there are no consequences to hypothetical decisions. Despite these shortcomings, there are steps that HIT investigators can take to engender more confidence in their survey responses. We recruited respondents from the public; however, developers and designers who are creating an HIT for a specific audience may want to recruit participants who are either in an at-risk demographic or actively seeking solutions in the HIT’s target domain. As realism is an important mediator in the intention–behavior gap, we also suggest that investigators craft their scenarios with the help of domain experts to make the scenarios as realistic as possible. Investigators could even add questions to their surveys that measure the degree to which respondents resonate with their scenarios; such measures could be used to either filter responses or create an additional modifying variable in the analyses.

HIT investigators may also want to consider focusing on short-term actions rather than long-term goals (eg, *I intend to eat more vegetables for dinner today* vs *I intend to lose 10 pounds this month*) when querying how a person would respond to an HIT; intention is believed to be a weaker predictor for long-term goals as completing them requires more self-efficacy and coordination to complete [76]. Finally, the health action process approach of Schwarzer [78] separates preintentional motivation and postintentional volition when measuring the likelihood of action; therefore, doing the same in health concept surveying may be beneficial.

To ensure that we were collecting meaningful responses, we also had to create plausible scenarios. We validated the scenarios used in our work through a pilot study using an abridged version of our survey instrument. Researchers who are investigating high-level questions as we did in our third case study would want to repeat this procedure; however, an HIT developer interested in advancing a particular HIT design while generating usable evidence may only need to assess scenario plausibility. We used a single question that explicitly asked respondents how plausible they believed a scenario to be; however, future researchers may want to investigate the nuances of plausibility through multiple questions. A person may believe a scenario is plausible as the health issue in question is common for their demographic or to people who engage in similar behaviors, or they may believe it is plausible because they do not have enough knowledge about the issue to know better. Researchers interested in examining HIT design decisions across multiple scenarios may also want to consider making their scenarios publicly available for future use. Sharing a common set of prevalidated scenarios would standardize the context of findings related to the same topic (eg, physical activity, step counting, and exercise).

### Conclusions

As more HITs transition from research to practice, it is important for HCI researchers to examine how those technologies will be received by the general population. Although one-off user studies provide actionable feedback for a specific HIT, they rarely provide insights that benefit other HIT creators. Our method, health concept surveying, attempts to strike a balance between actionable feedback and usable evidence. Using the HBM, health concept surveying disentangles proximal cognitive factors from HIT design decisions to explain *how* and *why* certain features are preferred. We used health concept surveying in 3 case studies to demonstrate the range of questions it can support and discussed the implications of the findings in each case. We hope that researchers will continue using health concept surveying in the future to better our understanding of HITs and accelerate their development.

## Appendix

Multimedia Appendix 1 An example of the health concept surveying survey instrument was applied to a design concept called SkinCheck.

Multimedia Appendix 2 A preliminary study was conducted to select the scenarios and design concepts that appear in this paper.

## Abbreviations

BCT: behavior change technology
CFI: comparative fit index
HBM: Health Belief Model
HCI: human–computer interaction
HIT: health intervention technology
IMC: instructional manipulation check
SRMR: standardized root mean square residual
