# Supplementary material for: Using Health Concept Surveying to Elicit Usable Evidence: Case Studies of a Novel Evaluation Methodology
Source: JMIR Hum Factors. 2022 Jan 3;9(1):e30474. doi: 10.2196/30474 (PMC8764610; doi:10.2196/30474)
Supplement: Multimedia Appendix 1 [file humanfactors_v9i1e30474_app1.docx]

**Multimedia Appendix 1. An example of the health concept surveying survey instrument was applied to a design concept called SkinCheck.**

*Note: To illustrate how the survey instrument is meant to be used, this instantiation uses skin cancer as the medical condition of interest. Other details that are meant to be completed by the researcher are indicated with double brackets (e.g.,* [[text]]*).*

# Consent

[[CONSENT DETAILS AS REQUIRED BY INSTITUTION]]

By clicking next, you agree:

- That you are at least 18 years of age,
- That you are participating in this study,
- That you understand you can withdraw from the survey at any time, and
- That you should refrain from providing identifiable data in open-ended questions.

**Required questions will be marked with an asterisk (*) sign.**

To begin the survey, **please click the “Next” button**.

# Exclusionary Criteria

1. What platform does your primary smartphone run on?

2. How would you rate your expertise with using a smartphone (e.g., using various apps, changing settings, etc.)?

# Survey Introduction

[[PURPOSE OF THE STUDY]]

**Disclaimer:** Any information you see in this survey (e.g., accuracy numbers, costs, diagnostic tests) should not be taken as medical advice as this information may not match the standard for any current or future procedures. Furthermore, this survey involves hypothetical scenarios with diagnostic aid smartphone apps that may not currently exist. For any medical concerns, consult your physician or doctor.

To confirm that you have read and understood the disclaimer above, please click the box below:

# Medical Condition Familiarity

3. How familiar, if at all, are you with skin cancer?

**Not familiar at all:** only heard the name, if at all
**Familiar:** familiar with some of the causes, symptoms, or treatments
**Extremely familiar:** familiar with all of the causes, symptoms, or treatments

| Not familiar at all | Slightly familiar | Somewhat familiar | Familiar | Moderately familiar | Very familiar | Extremely familiar |
| --- | --- | --- | --- | --- | --- | --- |
|  |  |  |  |  |  |  |

4. To the best of your knowledge, have you, a family member, or a close acquaintance had skin cancer in the past 3 years?

| Yes, I have or have had it in the past 3 years | Yes, a family member or close acquaintance has or has had it in the past 3 years | Yes, I and other family members or close acquaintances have or have had it in the past 3 years | No, neither me nor anybody I am close to has or has had it in the past 3 years | I am not sure | Prefer not to answer |
| --- | --- | --- | --- | --- | --- |
|  |  |  |  |  |  |

# Baseline HBM Measurements

Skin cancer is the most common form of cancer in the United States. Overexposure to ultraviolet (UV) light from the sun is the major cause of skin cancer. More information about the condition can be found at: <https://www.cdc.gov/cancer/skin/basic_info/what-is-skin-cancer.htm>.

5. If you had skin cancer, how much effect, if at all any, do you think it would have on your **long-term health**?

| No effect at all | Very little effect | Little effect | Moderate effect | Large effect | Very large effect | Extreme effect |
| --- | --- | --- | --- | --- | --- | --- |
|  |  |  |  |  |  |  |

6. If you had skin cancer, how much effect, if at all any, do you think it would have on your **finances**?

| No effect at all | Very little effect | Little effect | Moderate effect | Large effect | Very large effect | Extreme effect |
| --- | --- | --- | --- | --- | --- | --- |
|  |  |  |  |  |  |  |

7. If you had skin cancer, how much effect, if at all any, do you think it would have on you **socially and/or professionally**?

| No effect at all | Very little effect | Little effect | Moderate effect | Large effect | Very large effect | Extreme effect |
| --- | --- | --- | --- | --- | --- | --- |
|  |  |  |  |  |  |  |

8. How **beneficial**, if at all, do you think each of these actions would be towards improving your skin cancer?

|  | Not at all | Slightly beneficial | Somewhat beneficial | Beneficial | Moderately beneficial | Very beneficial | Extremely beneficial |
| --- | --- | --- | --- | --- | --- | --- | --- |
| Schedule an appointment with a doctor / physician |  |  |  |  |  |  |  |
| Contact a doctor's / physician's office for advice |  |  |  |  |  |  |  |

9. How **easy or difficult** do you think it would be for you to take each of the following actions?

|  | Very difficult | Somewhat difficult | Slightly difficult | Neither difficult nor easy | Slightly easy | Somewhat easy | Very easy |
| --- | --- | --- | --- | --- | --- | --- | --- |
| Schedule an appointment with a doctor / physician |  |  |  |  |  |  |  |
| Contact a doctor's / physician's office for advice |  |  |  |  |  |  |  |

# Scenario Description

**Imagine yourself in the following scenario and answer the following questions accordingly.**

You recently noticed a new mole (beauty mark) on your arm that is oddly colored and misshapen. After looking up information online, you worry that you might be developing skin cancer.

10. Please check all of the symptoms that were mentioned in the scenario you just read:

11. How likely or unlikely do you think you are to have skin cancer in this scenario?

| Very unlikely | Somewhat unlikely | Slightly unlikely | Neither unlikely nor likely | Slightly likely | Somewhat likely | Very likely |
| --- | --- | --- | --- | --- | --- | --- |
|  |  |  |  |  |  |  |

12. Given the possibility that you might have skin cancer, which of the following actions would you plan to take **on the same day** as when you discovered your symptoms?

|  | No / Probably No | Yes / Probably Yes |
| --- | --- | --- |
| Schedule an appointment with a doctor/physician |  |  |
| Contact a doctor's/physician's office for advice |  |  |

# App Description

A smartphone app named SkinCheck analyzes a picture of a mole to determine whether or not it is cancerous. To use the app, you are asked to take a picture of the mole so that it is clearly visible. The app guides you through taking a picture so that it can see the mole clearly and at a proper distance.

SkinCheck comes with your smartphone by default as part of a new mobile health initiative by [[RESPONDENT’S PHONE COMPANY]]. SkinCheck provides text-based and audio-based instructions to help you perform the test. The app also checks that the test was performed correctly. You can repeat the test until the app determines the image to be "valid". The results of the test are available instantly.

13. How likely or unlikely would you be to use SkinCheck to check your symptoms from the scenario?

| Very unlikely | Somewhat unlikely | Slightly unlikely | Neither unlikely nor likely | Slightly likely | Somewhat likely | Very likely |
| --- | --- | --- | --- | --- | --- | --- |
|  |  |  |  |  |  |  |

# “Positive” Test Result

*Note: This screen is only shown if the user’s answer to Question 13 was one of the following: “Neither unlikely nor likely”, “Slightly likely”, “Somewhat likely”, or “Very likely”.*

Imagine that you used SkinCheck and it said that the mark on your arm was **abnormal**. The app emphasizes that although it cannot guarantee that you have skin cancer, the test result suggests that you may want to seek medical care. With this test result in mind, please answer the same questions we asked you before.

14. How likely or unlikely do you think you are to have skin cancer in this scenario?

| Very unlikely | Somewhat unlikely | Slightly unlikely | Neither unlikely nor likely | Slightly likely | Somewhat likely | Very likely |
| --- | --- | --- | --- | --- | --- | --- |
|  |  |  |  |  |  |  |

15. Given the possibility that you might have skin cancer, which of the following actions would you plan to take **on the same day** as when you discovered your symptoms?

|  | No / Probably No | Yes / Probably Yes |
| --- | --- | --- |
| Schedule an appointment with a doctor/physician |  |  |
| Contact a doctor's/physician's office for advice |  |  |

# “Negative” Test Result

*Note: This screen is only shown if the user’s answer to Question 13 was one of the following: “Neither unlikely nor likely”, “Slightly likely”, “Somewhat likely”, or “Very likely”.*

Imagine that you used SkinCheck and it said that the mark on your arm was **normal**. The app emphasizes that although it cannot guarantee that you have skin cancer, the test result suggests that the mark on your arm is inconsistent with those of people with skin cancer. With this test result in mind, please answer the same questions we asked you before.

16. How likely or unlikely do you think you are to have skin cancer in this scenario?

| Very unlikely | Somewhat unlikely | Slightly unlikely | Neither unlikely nor likely | Slightly likely | Somewhat likely | Very likely |
| --- | --- | --- | --- | --- | --- | --- |
|  |  |  |  |  |  |  |

17. Given the possibility that you might have skin cancer, which of the following actions would you plan to take **on the same day** as when you discovered your symptoms?

|  | No / Probably No | Yes / Probably Yes |
| --- | --- | --- |
| Schedule an appointment with a doctor/physician |  |  |
| Contact a doctor's/physician's office for advice |  |  |

# Post-Survey Questionnaire Part 1

We have a few more questions before the survey is complete. Please answer the following questions so we can understand the factors that may have influenced your decisions.

18. In your opinion how would you rate **clinical tests**and **diagnostic aid apps** on the following factors?

|  | Very much so |  |  | No difference |  |  | Very much so |  |
| --- | --- | --- | --- | --- | --- | --- | --- | --- |
| I believe clinical tests are faster for getting results |  |  |  |  |  |  |  | I believe diagnostic aid apps are faster for getting results |
| I believe clinical tests are less expensive |  |  |  |  |  |  |  | I believe diagnostic aid apps are less expensive |
| I believe clinical tests keep records more private |  |  |  |  |  |  |  | I believe diagnostic aid apps keep records more private |

19. Can you think of any other information which might have changed the way you made your decisions in the previous scenarios?

20. How many courses have you taken involving statistics (pick the highest level)?

21. How often do you use statistics in your daily life?

22. Out of 1,000 people in a small town, 500 are members of a choir. Out of these 500 members in the choir, 100 are men. Out of the 500 inhabitants that are not in the choir, 300 are men. What is the probability that a randomly drawn man is a member of the choir? 

Please indicate the probability as a percentage without the percent sign (0-100).

# Post-Survey Questionnaire Part 2

Please answer the following demographic questions about yourself to the best of your abilities. Your answers will not be connected to any names, emails, or other personally-identifiable information.

23. What gender do you identify as?

24. What is your age?

25. What race and/or ethnicity do you identify as?

26. What is your current marital status?

27. Do you have any children?

28. In which country do you reside?

29. What is your highest level of education completed?

30. What is your current estimated annual household income?
