# Supplementary material for: Using Health Concept Surveying to Elicit Usable Evidence: Case Studies of a Novel Evaluation Methodology
Source: JMIR Hum Factors. 2022 Jan 3;9(1):e30474. doi: 10.2196/30474 (PMC8764610; doi:10.2196/30474)
Supplement: Multimedia Appendix 2 [file humanfactors_v9i1e30474_app2.docx]

**Multimedia Appendix 2. A preliminary study was conducted to select the scenarios and design concepts that appear in this paper.**

To demonstrate the flexibility of our method in a series of case studies, we first had to create a variety of prompts for plausible health-related scenarios and sensor-based health-screening apps. We describe the study we conducted to ensure that the hypotheticals that we would use in our case studies would be realistic.

### Study Design

In total, 96 respondents completed the survey from start to finish and passed the IMC. A subset of their demographic information is provided in Table I.

Table I. Demographic information for the people who completed the scenario and app selection survey.

| **Survey Demographics (N=96)** | |
| --- | --- |
| Source | Facebook (56), ITHS (37), Reddit (3) |
| Gender | Female (68), Male (26), Transgender Male (1), Gender Variant / Non-conforming (1) |
| Age | 18–24 (42), 25–34 (38), 35–44 (8), 45–54 (4),  55–64 (3), 65+ (1) |
| Smartphone Operating System | iOS (60), Android (36) |
| Self-Reported Smartphone Experience | Expert/Advanced (60), Intermediate (34), Novice/Beginner (2) |

Table II. The categories of medical conditions that were explored through the survey.

| **Category** | **Characteristics** | **Medical Conditions** | **Example** |
| --- | --- | --- | --- |
| Common | Relatively well-known; only requires short-term treatment; infectious | Sinus infection | Chandra et al. [1] |
|  |  | Strep throat | Nall and Charles [2] |
|  |  | Pink eye | Bhadra et al. [3] |
| Serious | Possibly fatal; requires long-term treatment | Pancreatic cancer | Mariakakis et al. [4] |
|  |  | Skin cancer | Wadhawan et al. [5] |
|  |  | Anemia | Wang et al. [6] |
| Stigmatizing | Could lead to uncomfortable social interactions if discovered by someone else | Halitosis | Seshan and Shwetha [7] |
|  |  | Irritable bowel syndrome | Lewis and Heaton [8] |
|  |  | Psoriasis | Shrivastava et al. [9] |

We created hypothetical scenarios and apps for three categories of medical conditions that we believed could elicit different reactions from people: *Common* conditions, *Serious* conditions, and *Stigmatizing* conditions. The categories are neither meant to be comprehensive nor definitive, but merely a formalized effort to explore different situations. We used two criteria that to pick the examples shown in Table II: (1) the condition had to involve a symptom that a person could theoretically perceive on their own to prompt investigation, and (2) the condition had to involve a symptom that could be detected with a sensor-based health-screening app using standard built-in smartphone sensors. Towards the latter point, each example we selected was inspired by an academic publication. Rather than rating the aforementioned outcome variables, respondents were asked to rate the plausibility of the scenarios (*ScenarioPlausibility*) and the plausibility of the apps (*AppPlausibility*) along a 7-point scale.

The survey was deployed in a 3×3 nested factorial design. The within-subject factor was the different categories of medical conditions (*ConditionType*), while the across-subject factor was the specific medical conditions within the categories (*Condition*). In other words, each respondent was randomly shown one medical condition from each category. The assignment of the conditions was counterbalanced, and the presentation order of the conditions in the survey was randomly shuffled. To determine the most representative medical conditions for each *ConditionType*, the HBM construct ratings were compared within the same category using the Kruskal-Wallis test [10]. When statistical significance was found, post-hoc Mann-Whitney U tests [11] with the Bonferroni-Holm correction [12] were used for pairwise comparisons. After the representative medical conditions were selected, a similar analysis was performed to compare HBM construct ratings across *ConditionType* to ensure that there was sufficient separation between them.

This survey had a completion rate of 68% when we account for respondents who ended the survey early, satisfied the exclusion criteria, or did not correctly answer the IMCs embedded in the survey. Ignoring cases when respondents took more than an hour-long break while completing the survey, the median survey completion time was 12 minutes.

### Results: Within Common Conditions

Statistically significant differences were found across the three *Common* conditions for both *ScenarioPlausibility* (*H*(2) = 9.091, *p* < .05) and *AppPlausibility* (*H*(2) = 8.247, *p* < .05). The sinus infection scenario was significantly less believable than the other two conditions (*p* < .05 versus both strep throat and pink eye). The pink eye app was significantly more believable than the sinus infection app (*p* < .05). Across all respondents who saw the pink eye scenario, 100% of them stated that the scenario was at least slightly believable, and 70% of them stated that the corresponding app was at least slightly believable. Given these results, we selected pink eye as our representative *Common* condition.

### Results: Within Serious Conditions

Statistically significant differences were found across the three *Serious* conditions for both *ScenarioPlausibility* (*H*(2) = 15.264, *p* < .001) and *AppPlausibility* (*H*(2) = 8.832, *p* < .05). The pancreatic cancer scenario was significantly less believable than the other two conditions (*p* < .01 versus both skin cancer and anemia). The skin cancer app was significantly more believable than the anemia app (*p* < .01). Across all respondents who saw the skin cancer scenario, 93% of them stated that the scenario was at least slightly believable, and 80% of the respondents stated that the corresponding app was at least slightly believable. Given these results, we selected skin cancer as our representative *Serious* condition.

### Results: Within Stigmatizing Conditions

A statistically significant difference was only found across the three *Stigmatizing* conditions for *AppPlausibility* (*H*(2) = 6.420, *p* < .05). The psoriasis app was slightly more believable than the IBS app (*p* = .06). However, there was a statistically significant difference between the three *Stigmatizing* conditions regarding the impact they would have on a person's social life and professional standing (*H*(2) = 14.892, *p* < .01). In particular, psoriasis was deemed significantly less impactful than the other two conditions (*p* < .01 versus both halitosis and IBS). Since halitosis was rated at least as high as the other *Stigmatizing* conditions in terms of *ScenarioPlausibility*, *AppPlausibility*, and *PerceivedSeriousness*, we selected halitosis as our representative *Stigmatizing* condition. Across all respondents who saw the halitosis scenario, 90% of them stated that the scenario was at least slightly believable, and 47% of them stated that the corresponding app was at least slightly believable. Although the latter number is low compared to the other condition categories, only 27% of the respondents said that the halitosis app was unbelievable to some degree.

### Results: Across Condition Types

Figure I. The distribution of ratings for (left) *ScenarioPlausibility* and (right) *AppPlausibility*.


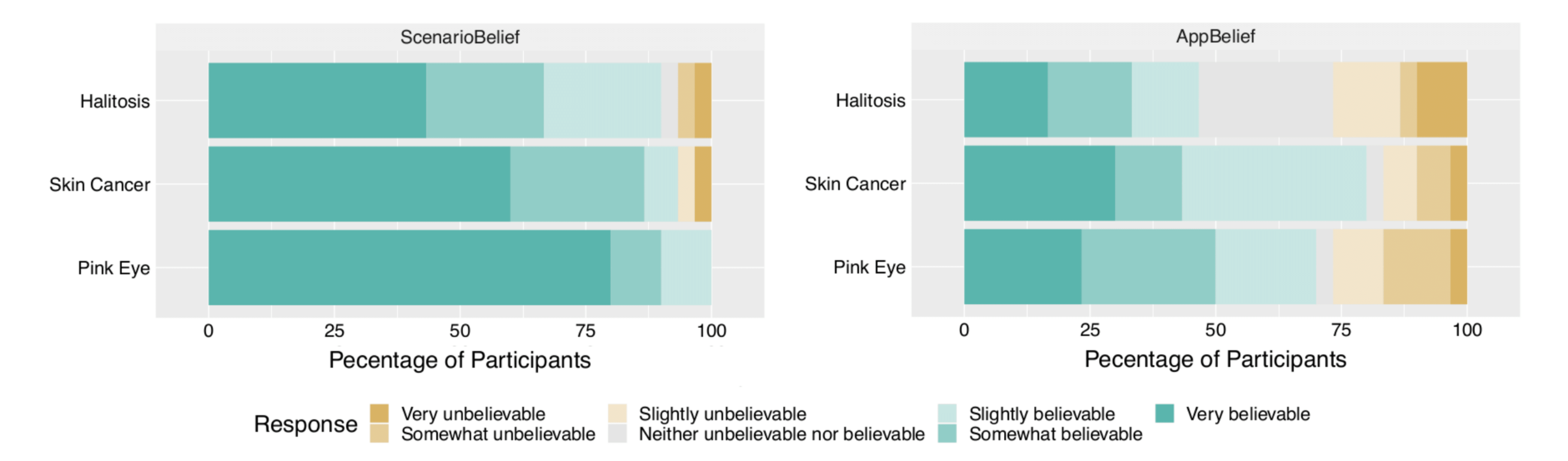


Figure I shows the distribution of *ScenarioPlausibility* and *AppPlausibility* ratings for the three selected medical conditions. The conditions were not significantly different according to *AppPlausibility* (*H*(2) = 3.067, *n.s.*). According to *ScenarioPlausibility*, however, they were different (*H*(2) = 9.068, *p* < .05), with the pink eye scenario being more believable than the scenario about halitosis (*p* < .01). Nevertheless, we were satisfied with the selected conditions since they all had high median *ScenarioPlausibility* ratings. Statistically significant differences were found across the three conditions for all of the HBM constructs, including *PerceivedSeriousness* regarding long-term health (*H*(2) = 47.352, *p* < .001), *PerceivedSeriousness* regarding finances (*H*(2) = 49.162, *p* < .001), *PerceivedSeriousness* regarding social standing (*H*(2) = 16.128, *p* < .001), and *PerceivedSusceptibility* (*H*(2) = 34.218, *p* < .001). There were no statistically significant ordering effects for these tests.

Our definition of a *Serious* medical condition suggests that skin cancer should have a higher impact on a person's long-term health and finances than the other two medical conditions. The definition also suggests that people should believe that they are less prone to having skin cancer than the other medical conditions. Our results supported both hypotheses. Skin cancer was rated as having a significantly higher *PerceivedSeriousness* regarding long-term health (*p* < .001), higher *PerceivedSeriousness* regarding finances (*p* < .001), and lower *PerceivedSusceptibility* (*p* < .001) compared to pink eye and halitosis. Our definition of a *Stigmatizing* medical condition suggests that halitosis should have a higher impact on a person's social life and professional standing than the other two medical conditions. Halitosis was rated as having a significantly higher *PerceivedSeriousness* on social standing than pink eye (*p* < .05); however, there was not a significant difference between halitosis and skin cancer. Nevertheless, the other characteristics that were unique to skin cancer as a *Serious* condition provided enough separation between them. The combination of these results indicates that pink eye was viewed as having low *PerceivedSeriousness* and high *PerceivedSusceptibility*. Therefore, pink eye was deemed suitable as a *Common* condition.

## References

1. Chandra RK, Patadia MO, Raviv J. Diagnosis of Nasal Airway Obstruction. Otolaryngol Clin North Am [Internet] 2009 [cited 2019 Mar 27];42(2):207–225. [doi: 10.1016/j.otc.2009.01.004]

2. Nall R, Charles M. Is it strep throat? Pictures and symptoms [Internet]. 2017 [cited 2019 Apr 8]. Available from: https://www.medicalnewstoday.com/articles/312433.php

3. Bhadra AA, Jain M, Shidnal S. Automated detection of eye diseases. Proc 2016 IEEE Int Conf Wirel Commun Signal Process Networking, WiSPNET 2016 [Internet] IEEE; 2016 [cited 2019 Mar 27]. p. 1341–1345. [doi: 10.1109/WiSPNET.2016.7566355]

4. Mariakakis A, Banks MA, Phillipi L, Yu L, Taylor J, Patel SN. BiliScreen: Smartphone-Based Scleral Jaundice Monitoring for Liver and Pancreatic Disorders. Proc IMWUT ’17 [Internet] 2017 [cited 2017 Dec 4];1(2):20:1-20:26. [doi: 10.1145/3090085]

5. Wadhawan T, Situ N, Rui H, Lancaster K, Yuan X, Zouridakis G. Implementation of the 7-point checklist for melanoma detection on smart handheld devices. Proc Annu Int Conf IEEE Eng Med Biol Soc EMBS [Internet] IEEE; 2011 [cited 2017 Feb 28]. p. 3180–3183. PMID:22255015

6. Wang EJ, Li W, Hawkins D, Gernsheimer T, Norby-Slycord C, Patel SN. HemaApp: Noninvasive Blood Screening of Hemoglobin Using Smartphone Cameras. Proc 2016 ACM Int Jt Conf Pervasive Ubiquitous Comput - UbiComp ’16 [Internet] 2016 [cited 2017 Jul 14]. p. 593–604. [doi: 10.1145/2971648.2971653]

7. Seshan H, Shwetha M. Gingival inflammation assessment: Image analysis. J Indian Soc Periodontol [Internet] Wolters Kluwer -- Medknow Publications; 2012 Apr [cited 2019 Mar 28];16(2):231. PMID:23055590

8. Lewis SJ, Heaton KW. Stool form scale as a useful guide to intestinal transit time. Scand J Gastroenterol [Internet] Taylor & Francis; 1997 Jan 8 [cited 2019 Apr 8];32(9):920–924. [doi: 10.3109/00365529709011203]

9. Shrivastava VK, Londhe ND, Sonawane RS, Suri JS. Computer-aided diagnosis of psoriasis skin images with HOS, texture and color features: A first comparative study of its kind. Comput Methods Programs Biomed [Internet] Elsevier; 2016 Apr 1 [cited 2019 Mar 27];126:98–109. [doi: 10.1016/J.CMPB.2015.11.013]

10. Kruskal WH, Wallis WA. Use of ranks in one-criteron analysis of variance. J Am Stat Assoc [Internet] 1952 [cited 2018 Aug 9];47(260):583–621. [doi: 10.2307/2280779]

11. Mann HB, Whitney DR. On a Test of Whether one of Two Random Variables is Stochastically Larger than the Other. Ann Math Stat [Internet] 1947 [cited 2018 Jun 5];18(1):50–60. PMID:10226

12. Holm S. A Simple Sequentially Rejective Multiple Test Procedure. Scand J Stat [Internet] 1979 [cited 2018 Aug 8];6:65–70. PMID:442
